# 1,1′-[1,4-Phenyl­enebis(methyl­ene)]bis­(2-methyl-1*H*-imidazol-3-ium) 2,4-dicarb­oxy­benzene-1,5-dicarboxyl­ate monohydrate

**DOI:** 10.1107/S160053681102263X

**Published:** 2011-06-18

**Authors:** Gui-Ying Dong, Tong-Fei Liu, Cui-Hong He, Xiao-Chen Deng, Xiao-Ge Shi

**Affiliations:** a College of Chemical Engineering, Hebei United University, Tangshan 063009, People’s Republic of China; b Qian’an College, Hebei United University, Tangshan 063009, People’s Republic of China

## Abstract

In the dication of the title compound, C_16_H_20_N_4_^2+^·C_10_H_4_O_8_^2−^·H_2_O, the dihedral angles formed by mean planes of the imidazolium rings and the benzene ring are 69.05 (18) and 89.1 (2)°. In the crystal, the components are linked into a three-dimensional network by inter­molecular N—H⋯O and O—H⋯O hydrogen bonds.

## Related literature

For the synthesis of 1,4-bis­(2-methyl-1*H*-imidazole-3-ium)benzene, see: Hoskins *et al.* (1997 ▶). For related complexes, see: Liu Wu, Wan *et al.* (2011 ▶); Liu, Wu, Zhang & Cui (2011 ▶). 
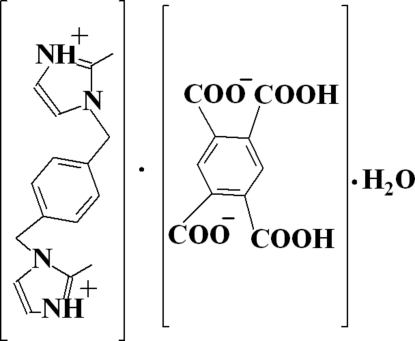


## Experimental

### 

#### Crystal data

C_16_H_20_N_4_^2+^·C_10_H_4_O_8_^2−^·H_2_O
*M* *_r_* = 538.51
Monoclinic, 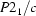

*a* = 9.7139 (19) Å
*b* = 19.428 (4) Å
*c* = 13.856 (3) Å
β = 97.39 (3)°
*V* = 2593.3 (9) Å^3^
*Z* = 4
Mo *K*α radiation
μ = 0.11 mm^−1^
*T* = 295 K
0.22 × 0.18 × 0.16 mm

#### Data collection

Bruker SMART CCD area-detector diffractometer
Absorption correction: multi-scan (*SADABS*; Sheldrick, 1996 ▶) *T*_min_ = 0.968, *T*_max_ = 0.971
20668 measured reflections
4571 independent reflections
3075 reflections with *I* > 2σ(*I*)
*R*_int_ = 0.078

#### Refinement

*R*[*F* ^2^ > 2σ(*F* ^2^)] = 0.073
*wR*(*F* ^2^) = 0.144
*S* = 1.10
4571 reflections
356 parameters
H-atom parameters constrained
Δρ_max_ = 0.30 e Å^−3^
Δρ_min_ = −0.25 e Å^−3^

### 

Data collection: *SMART* (Bruker, 1998 ▶); cell refinement: *SAINT* (Bruker, 1999 ▶); data reduction: *SAINT*; program(s) used to solve structure: *SHELXS97* (Sheldrick, 2008 ▶); program(s) used to refine structure: *SHELXL97* (Sheldrick, 2008 ▶); molecular graphics: *SHELXTL* (Sheldrick, 2008 ▶); software used to prepare material for publication: *SHELXTL*.

## Supplementary Material

Crystal structure: contains datablock(s) I, global. DOI: 10.1107/S160053681102263X/lh5268sup1.cif

Structure factors: contains datablock(s) I. DOI: 10.1107/S160053681102263X/lh5268Isup2.hkl

Supplementary material file. DOI: 10.1107/S160053681102263X/lh5268Isup3.cml

Additional supplementary materials:  crystallographic information; 3D view; checkCIF report

## Figures and Tables

**Table 1:** Hydrogen-bond geometry (Å, °)

*D*—H⋯*A*	*D*—H	H⋯*A*	*D*⋯*A*	*D*—H⋯*A*
O1—H1⋯O6^i^	0.82	1.76	2.575 (3)	172
O1*W*—H1*A*⋯O6	0.86	1.90	2.747 (3)	168
O1*W*—H1*B*⋯O5^ii^	0.85	1.98	2.743 (3)	149
N4—H1*C*⋯O4^iii^	0.98	1.75	2.679 (4)	158
N2—H2⋯O1*W*^iv^	1.07	1.59	2.651 (4)	172
O8—H8⋯O3^v^	0.82	1.81	2.586 (3)	157

Symmetry codes: (i) 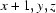; (ii) 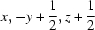; (iii) 
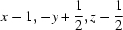
; (iv) 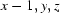; (v) 
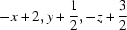
.

**Fig. 1.**
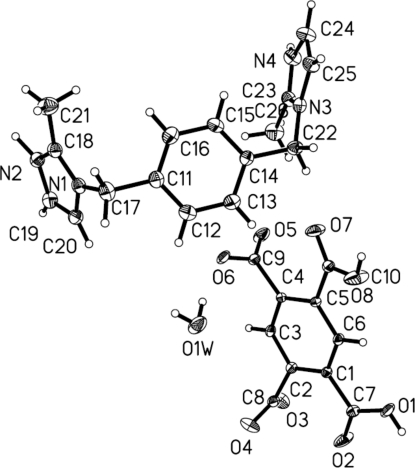
Fig. 1. The molecular structure of (I), showing displacement ellipsoids at the 30% probability levels.

## supplementary crystallographic information

### Comment

1,4-bis(2-methylimidazole-1-methyl)benzene is a flexible bridging ligand, which can participate in the construction of coordination polymers and can provide information on the influences of 2-substituted derivatives of imidazole on the structures and properties of resulting complexes (Liu *et al.*, 2011*a*,2011*b*). In our attempt to synthesize a zinc complex with this ligand, we unexpectedly obtained the title compound (I) and report herein its synthesis and crystal structure determination.

The asymmetric unit of (I) is shown in (Fig. 1). Bond distances and angles are as found in other structures (Liu Wu, Wan *et al.*, 2011); Liu, Wu, Zhang & Cui, 2011). In the dication, the dihedral angles formed by the mean-planes of the imidazole rings and the benzene ring (C11-C16) are 69.05 (18)° (N1/N2/C18/C19/C20) and 89.1 (2)° (N3/N4/C23/C24/C25). In the crystal, the components are linked into a three-dimensional network by intermolecular N—H···O and O—H···O hydrogen bonds.

### Experimental

1,4-Bis(2-methylimidazole-1-methyl)benzene was prepared by the method of Hoskins *et al.* (1997). A mixture of Zn(NO_3_)_2_.6H_2_O (298 mg,1 mmol), 1,4-bis(2-methylimidazole-1-methyl)benzene (1.0 mmol, 266.4 mg), 1,2,4,5-benzenetetracarboxylic acid (1.0 mmol, 254.2 mg) and H_2_O (12 mL) was placed in a Teflon-lined stainless vessel and heated to 393 K for 4 days under autogenous pressure, and then cooled to room temperature in 24 h. block-shaped colourless crystals of (I) suitable for single-crystal X-ray diffraction analysis were obtained.

### Refinement

H atoms bonded to C atoms and hydroxy H atoms were placed in calculated positions with C–H = 0.93 or 0.97 Å, O-H = 0.82Å and refined using a riding-model approximation with *U*_iso_(H) = 1.2*U*_eq_(C) or *U*_iso_(H) = 1.5*U*_eq_(O, C_methyl_). Water H atoms and N-bound H atoms were located in a difference Fourier map and included in their 'as found' positions with *U*_iso_(H) = 1.5*U*_eq_(O, N).

### Crystal data

**Table:**

C_16_H_20_N_4_^2+^·C_10_H_4_O_8_^2^^−^·H_2_O	*F*(000) = 1128
*M**_r_* = 538.51	*D*_x_ = 1.379 Mg m^−^^3^
Monoclinic, *P*2_1_/*c*	Mo *K*α radiation, λ = 0.71073 Å
Hall symbol: -P 2ybc	Cell parameters from 2963 reflections
*a* = 9.7139 (19) Å	θ = 4.6–23.5°
*b* = 19.428 (4) Å	µ = 0.11 mm^−^^1^
*c* = 13.856 (3) Å	*T* = 295 K
β = 97.39 (3)°	Block, colourless
*V* = 2593.3 (9) Å^3^	0.22 × 0.18 × 0.16 mm
*Z* = 4	

### Data collection

**Table:**

Bruker SMART CCD area-detector diffractometer	4571 independent reflections
Radiation source: fine–focus sealed tube	3075 reflections with *I* > 2σ(*I*)
graphite	*R*_int_ = 0.078
φ and ω scans	θ_max_ = 25.0°, θ_min_ = 3.0°
Absorption correction: multi-scan (*SADABS*; Sheldrick, 1996)	*h* = −11→11
*T*_min_ = 0.968, *T*_max_ = 0.971	*k* = −23→23
20668 measured reflections	*l* = −16→16

### Refinement

**Table:**

Refinement on *F*^2^	Primary atom site location: structure-invariant direct methods
Least-squares matrix: full	Secondary atom site location: difference Fourier map
*R*[*F*^2^ > 2σ(*F*^2^)] = 0.073	Hydrogen site location: inferred from neighbouring sites
*wR*(*F*^2^) = 0.144	H-atom parameters constrained
*S* = 1.10	*w* = 1/[σ^2^(*F*_o_^2^) + (0.0452*P*)^2^ + 1.9004*P*] where *P* = (*F*_o_^2^ + 2*F*_c_^2^)/3
4571 reflections	(Δ/σ)_max_ = 0.001
356 parameters	Δρ_max_ = 0.30 e Å^−^^3^
0 restraints	Δρ_min_ = −0.25 e Å^−^^3^

### Special details

**Table:**

Geometry. All e.s.d.'s (except the e.s.d. in the dihedral angle between two l.s. planes) are estimated using the full covariance matrix. The cell e.s.d.'s are taken into account individually in the estimation of e.s.d.'s in distances, angles and torsion angles; correlations between e.s.d.'s in cell parameters are only used when they are defined by crystal symmetry. An approximate (isotropic) treatment of cell e.s.d.'s is used for estimating e.s.d.'s involving l.s. planes.
Refinement. Refinement of *F*^2^ against ALL reflections. The weighted *R*-factor *wR* and goodness of fit *S* are based on *F*^2^, conventional *R*-factors *R* are based on *F*, with *F* set to zero for negative *F*^2^. The threshold expression of *F*^2^ > σ(*F*^2^) is used only for calculating *R*-factors(gt) *etc*. and is not relevant to the choice of reflections for refinement. *R*-factors based on *F*^2^ are statistically about twice as large as those based on *F*, and *R*- factors based on ALL data will be even larger.

### Fractional atomic coordinates and isotropic or equivalent isotropic displacement parameters (Å^2^)

**Table:**

	*x*	*y*	*z*	*U*_iso_*/*U*_eq_	
O1	1.3527 (2)	0.26814 (12)	0.7753 (2)	0.0460 (7)	
H1	1.4361	0.2598	0.7806	0.069*	
C1	1.1278 (3)	0.21981 (14)	0.7695 (2)	0.0211 (7)	
O6	0.6182 (2)	0.25426 (12)	0.78948 (16)	0.0389 (6)	
N2	0.0283 (3)	0.27283 (15)	1.00182 (19)	0.0370 (7)	
C6	1.0713 (3)	0.28578 (15)	0.7517 (2)	0.0241 (7)	
H6	1.1309	0.3232	0.7512	0.029*	
C5	0.9277 (3)	0.29696 (14)	0.7346 (2)	0.0215 (7)	
C2	1.0382 (3)	0.16286 (15)	0.7718 (2)	0.0228 (7)	
O3	1.0942 (3)	0.04591 (11)	0.73740 (17)	0.0466 (7)	
C10	0.8718 (3)	0.36897 (15)	0.7201 (2)	0.0280 (7)	
O7	0.7529 (2)	0.38125 (12)	0.6872 (2)	0.0552 (8)	
C4	0.8365 (3)	0.23966 (15)	0.7314 (2)	0.0211 (7)	
C9	0.6773 (3)	0.24479 (15)	0.7138 (2)	0.0280 (7)	
C3	0.8944 (3)	0.17407 (15)	0.7510 (2)	0.0251 (7)	
H3	0.8351	0.1364	0.7501	0.030*	
N3	0.4318 (3)	0.51795 (13)	0.63373 (19)	0.0311 (7)	
O5	0.6155 (2)	0.23446 (14)	0.62994 (17)	0.0487 (7)	
N1	0.2118 (3)	0.33609 (14)	1.03805 (19)	0.0341 (7)	
C7	1.2841 (3)	0.21099 (16)	0.7850 (2)	0.0269 (7)	
O4	1.1014 (3)	0.07904 (12)	0.89343 (18)	0.0526 (7)	
C18	0.0723 (4)	0.33800 (18)	1.0185 (2)	0.0359 (8)	
O2	1.3390 (2)	0.15570 (12)	0.8038 (2)	0.0622 (9)	
C8	1.0850 (3)	0.08958 (16)	0.8028 (3)	0.0291 (8)	
C14	0.4739 (3)	0.47375 (16)	0.8037 (2)	0.0303 (8)	
O8	0.9640 (3)	0.41682 (12)	0.7470 (3)	0.0736 (10)	
H8	0.9268	0.4547	0.7415	0.110*	
C23	0.3679 (4)	0.47019 (17)	0.5734 (2)	0.0355 (8)	
C25	0.3698 (4)	0.58156 (17)	0.6113 (3)	0.0398 (9)	
H25	0.3942	0.6232	0.6419	0.048*	
C17	0.3073 (4)	0.39578 (18)	1.0599 (3)	0.0445 (9)	
H17A	0.3840	0.3821	1.1079	0.053*	
H17B	0.2579	0.4327	1.0877	0.053*	
C11	0.3644 (4)	0.42225 (17)	0.9695 (2)	0.0369 (9)	
N4	0.2683 (3)	0.50177 (15)	0.5137 (2)	0.0436 (8)	
C22	0.5378 (3)	0.50329 (17)	0.7177 (2)	0.0357 (8)	
H22A	0.5868	0.5454	0.7377	0.043*	
H22B	0.6047	0.4708	0.6981	0.043*	
C19	0.1416 (4)	0.22940 (18)	1.0100 (3)	0.0411 (9)	
H19	0.1395	0.1819	1.0015	0.049*	
C20	0.2568 (4)	0.26881 (18)	1.0327 (2)	0.0388 (9)	
H20	0.3482	0.2535	1.0427	0.047*	
C13	0.5502 (4)	0.4301 (2)	0.8693 (3)	0.0532 (11)	
H13	0.6392	0.4174	0.8586	0.064*	
C24	0.2675 (4)	0.57139 (19)	0.5368 (3)	0.0476 (10)	
H24	0.2079	0.6047	0.5068	0.057*	
C16	0.2873 (5)	0.4638 (2)	0.9035 (3)	0.0718 (14)	
H16	0.1973	0.4755	0.9134	0.086*	
C12	0.4964 (4)	0.4048 (2)	0.9512 (3)	0.0556 (11)	
H12	0.5504	0.3758	0.9940	0.067*	
C15	0.3412 (4)	0.4890 (2)	0.8213 (3)	0.0677 (14)	
H15	0.2857	0.5167	0.7775	0.081*	
O1W	0.7588 (2)	0.24523 (16)	0.97416 (18)	0.0651 (9)	
C26	0.4009 (4)	0.39518 (18)	0.5750 (3)	0.0522 (11)	
H26A	0.3520	0.3725	0.6219	0.078*	
H26B	0.4990	0.3889	0.5923	0.078*	
H26C	0.3731	0.3759	0.5117	0.078*	
C21	−0.0224 (4)	0.3989 (2)	1.0153 (3)	0.0587 (11)	
H21A	−0.0020	0.4250	1.0742	0.088*	
H21B	−0.1170	0.3835	1.0089	0.088*	
H21C	−0.0089	0.4274	0.9606	0.088*	
H1C	0.2174	0.4787	0.4575	0.088*	
H2	−0.0820	0.2658	0.9924	0.088*	
H1A	0.7048	0.2485	0.9205	0.088*	
H1B	0.6895	0.2529	1.0044	0.088*	

### Atomic displacement parameters (Å^2^)

**Table:**

	*U*^11^	*U*^22^	*U*^33^	*U*^12^	*U*^13^	*U*^23^
O1	0.0160 (11)	0.0409 (15)	0.0811 (19)	0.0003 (11)	0.0055 (13)	0.0144 (13)
C1	0.0194 (16)	0.0217 (16)	0.0228 (17)	0.0016 (13)	0.0051 (13)	−0.0015 (13)
O6	0.0169 (11)	0.0709 (17)	0.0295 (13)	0.0036 (11)	0.0052 (10)	0.0011 (12)
N2	0.0357 (17)	0.0423 (18)	0.0331 (17)	0.0002 (14)	0.0045 (14)	−0.0022 (13)
C6	0.0203 (16)	0.0203 (16)	0.0315 (18)	−0.0035 (13)	0.0025 (14)	0.0007 (13)
C5	0.0186 (16)	0.0220 (16)	0.0241 (17)	0.0036 (13)	0.0037 (13)	0.0022 (13)
C2	0.0222 (16)	0.0205 (16)	0.0258 (17)	0.0003 (13)	0.0034 (13)	−0.0019 (13)
O3	0.0692 (18)	0.0224 (13)	0.0476 (16)	0.0054 (12)	0.0056 (13)	−0.0041 (12)
C10	0.0247 (18)	0.0238 (18)	0.035 (2)	0.0001 (15)	0.0030 (15)	0.0039 (14)
O7	0.0304 (15)	0.0345 (15)	0.097 (2)	0.0101 (11)	−0.0081 (14)	0.0086 (14)
C4	0.0175 (15)	0.0250 (17)	0.0206 (16)	0.0013 (13)	0.0016 (13)	0.0034 (13)
C9	0.0210 (17)	0.0278 (18)	0.035 (2)	0.0005 (14)	0.0019 (16)	0.0033 (15)
C3	0.0207 (16)	0.0234 (17)	0.0312 (18)	−0.0071 (13)	0.0029 (14)	0.0013 (14)
N3	0.0331 (15)	0.0300 (16)	0.0306 (16)	−0.0048 (13)	0.0059 (13)	−0.0040 (13)
O5	0.0243 (13)	0.086 (2)	0.0335 (15)	−0.0021 (13)	−0.0040 (11)	−0.0098 (13)
N1	0.0387 (17)	0.0352 (17)	0.0283 (16)	−0.0049 (14)	0.0037 (13)	0.0000 (13)
C7	0.0249 (17)	0.0249 (18)	0.0303 (19)	0.0020 (15)	0.0015 (15)	−0.0041 (14)
O4	0.0766 (19)	0.0400 (15)	0.0376 (16)	0.0118 (13)	−0.0067 (14)	0.0096 (12)
C18	0.041 (2)	0.040 (2)	0.0267 (19)	0.0029 (17)	0.0053 (16)	−0.0015 (16)
O2	0.0251 (13)	0.0291 (14)	0.130 (3)	0.0091 (11)	−0.0012 (15)	0.0043 (15)
C8	0.0228 (17)	0.0228 (18)	0.041 (2)	−0.0009 (14)	0.0020 (16)	0.0040 (16)
C14	0.0293 (18)	0.0299 (18)	0.0314 (19)	−0.0041 (15)	0.0023 (15)	−0.0035 (15)
O8	0.0444 (16)	0.0205 (14)	0.146 (3)	0.0020 (12)	−0.0264 (18)	0.0019 (17)
C23	0.040 (2)	0.032 (2)	0.034 (2)	−0.0040 (16)	0.0042 (17)	−0.0010 (16)
C25	0.049 (2)	0.0268 (19)	0.043 (2)	−0.0006 (17)	0.0027 (19)	−0.0008 (17)
C17	0.052 (2)	0.043 (2)	0.038 (2)	−0.0125 (19)	0.0045 (18)	−0.0023 (17)
C11	0.040 (2)	0.033 (2)	0.038 (2)	−0.0066 (17)	0.0048 (17)	−0.0026 (16)
N4	0.0488 (19)	0.0444 (19)	0.0342 (18)	−0.0055 (15)	−0.0076 (15)	−0.0051 (14)
C22	0.0316 (19)	0.036 (2)	0.038 (2)	−0.0015 (15)	−0.0029 (16)	−0.0011 (16)
C19	0.048 (2)	0.034 (2)	0.042 (2)	−0.0005 (18)	0.0113 (18)	−0.0044 (17)
C20	0.040 (2)	0.041 (2)	0.037 (2)	0.0061 (17)	0.0092 (17)	0.0013 (17)
C13	0.043 (2)	0.061 (3)	0.059 (3)	0.016 (2)	0.015 (2)	0.015 (2)
C24	0.055 (3)	0.040 (2)	0.044 (2)	0.0078 (19)	−0.006 (2)	0.0033 (18)
C16	0.055 (3)	0.093 (4)	0.073 (3)	0.030 (3)	0.034 (2)	0.032 (3)
C12	0.050 (3)	0.061 (3)	0.057 (3)	0.014 (2)	0.010 (2)	0.021 (2)
C15	0.055 (3)	0.084 (3)	0.068 (3)	0.030 (2)	0.022 (2)	0.045 (3)
O1W	0.0330 (14)	0.123 (3)	0.0385 (16)	−0.0012 (15)	0.0002 (12)	−0.0064 (16)
C26	0.059 (3)	0.036 (2)	0.059 (3)	−0.0044 (18)	0.000 (2)	−0.0091 (19)
C21	0.059 (3)	0.054 (3)	0.063 (3)	0.014 (2)	0.007 (2)	−0.004 (2)

### Geometric parameters (Å, °)

**Table:**

O1—C7	1.311 (4)	C14—C22	1.524 (5)
O1—H1	0.8200	O8—H8	0.8200
C1—C6	1.404 (4)	C23—N4	1.338 (4)
C1—C2	1.411 (4)	C23—C26	1.492 (5)
C1—C7	1.516 (4)	C25—C24	1.352 (5)
O6—C9	1.272 (4)	C25—H25	0.9300
N2—C18	1.347 (4)	C17—C11	1.523 (5)
N2—C19	1.380 (4)	C17—H17A	0.9700
N2—H2	1.0718	C17—H17B	0.9700
C6—C5	1.402 (4)	C11—C16	1.369 (5)
C6—H6	0.9300	C11—C12	1.381 (5)
C5—C4	1.420 (4)	N4—C24	1.390 (4)
C5—C10	1.505 (4)	N4—H1C	0.9761
C2—C3	1.406 (4)	C22—H22A	0.9700
C2—C8	1.539 (4)	C22—H22B	0.9700
O3—C8	1.253 (4)	C19—C20	1.359 (5)
C10—O7	1.210 (4)	C19—H19	0.9300
C10—O8	1.312 (4)	C20—H20	0.9300
C4—C3	1.405 (4)	C13—C12	1.398 (5)
C4—C9	1.538 (4)	C13—H13	0.9300
C9—O5	1.253 (4)	C24—H24	0.9300
C3—H3	0.9300	C16—C15	1.402 (5)
N3—C23	1.346 (4)	C16—H16	0.9300
N3—C25	1.392 (4)	C12—H12	0.9300
N3—C22	1.479 (4)	C15—H15	0.9300
N1—C18	1.349 (4)	O1W—H1A	0.8558
N1—C20	1.383 (4)	O1W—H1B	0.8509
N1—C17	1.492 (4)	C26—H26A	0.9600
C7—O2	1.212 (4)	C26—H26B	0.9600
O4—C8	1.262 (4)	C26—H26C	0.9600
C18—C21	1.496 (5)	C21—H21A	0.9600
C14—C15	1.374 (5)	C21—H21B	0.9600
C14—C13	1.386 (5)	C21—H21C	0.9600
			
C7—O1—H1	109.5	N1—C17—C11	112.2 (3)
C6—C1—C2	119.5 (3)	N1—C17—H17A	109.2
C6—C1—C7	119.4 (3)	C11—C17—H17A	109.2
C2—C1—C7	121.2 (3)	N1—C17—H17B	109.2
C18—N2—C19	109.2 (3)	C11—C17—H17B	109.2
C18—N2—H2	115.5	H17A—C17—H17B	107.9
C19—N2—H2	134.9	C16—C11—C12	117.6 (3)
C5—C6—C1	122.0 (3)	C16—C11—C17	121.5 (3)
C5—C6—H6	119.0	C12—C11—C17	120.9 (3)
C1—C6—H6	119.0	C23—N4—C24	109.2 (3)
C6—C5—C4	119.2 (3)	C23—N4—H1C	122.3
C6—C5—C10	120.1 (3)	C24—N4—H1C	127.8
C4—C5—C10	120.8 (2)	N3—C22—C14	112.0 (3)
C3—C2—C1	118.2 (3)	N3—C22—H22A	109.2
C3—C2—C8	116.8 (2)	C14—C22—H22A	109.2
C1—C2—C8	124.8 (3)	N3—C22—H22B	109.2
O7—C10—O8	123.5 (3)	C14—C22—H22B	109.2
O7—C10—C5	123.0 (3)	H22A—C22—H22B	107.9
O8—C10—C5	113.5 (3)	C20—C19—N2	107.4 (3)
C3—C4—C5	118.2 (3)	C20—C19—H19	126.3
C3—C4—C9	117.3 (3)	N2—C19—H19	126.3
C5—C4—C9	124.4 (2)	C19—C20—N1	106.8 (3)
O5—C9—O6	125.1 (3)	C19—C20—H20	126.6
O5—C9—C4	119.2 (3)	N1—C20—H20	126.6
O6—C9—C4	115.4 (3)	C14—C13—C12	121.6 (3)
C4—C3—C2	122.9 (3)	C14—C13—H13	119.2
C4—C3—H3	118.6	C12—C13—H13	119.2
C2—C3—H3	118.6	C25—C24—N4	107.0 (3)
C23—N3—C25	108.8 (3)	C25—C24—H24	126.5
C23—N3—C22	125.1 (3)	N4—C24—H24	126.5
C25—N3—C22	125.7 (3)	C11—C16—C15	121.3 (4)
C18—N1—C20	109.4 (3)	C11—C16—H16	119.4
C18—N1—C17	127.0 (3)	C15—C16—H16	119.4
C20—N1—C17	123.6 (3)	C11—C12—C13	121.0 (4)
O2—C7—O1	123.9 (3)	C11—C12—H12	119.5
O2—C7—C1	122.4 (3)	C13—C12—H12	119.5
O1—C7—C1	113.8 (3)	C14—C15—C16	121.7 (4)
N2—C18—N1	107.3 (3)	C14—C15—H15	119.1
N2—C18—C21	124.0 (3)	C16—C15—H15	119.1
N1—C18—C21	128.8 (3)	H1A—O1W—H1B	88.8
O3—C8—O4	126.8 (3)	C23—C26—H26A	109.5
O3—C8—C2	118.0 (3)	C23—C26—H26B	109.5
O4—C8—C2	115.0 (3)	H26A—C26—H26B	109.5
C15—C14—C13	116.7 (3)	C23—C26—H26C	109.5
C15—C14—C22	122.9 (3)	H26A—C26—H26C	109.5
C13—C14—C22	120.4 (3)	H26B—C26—H26C	109.5
C10—O8—H8	109.5	C18—C21—H21A	109.5
N4—C23—N3	107.9 (3)	C18—C21—H21B	109.5
N4—C23—C26	126.4 (3)	H21A—C21—H21B	109.5
N3—C23—C26	125.7 (3)	C18—C21—H21C	109.5
C24—C25—N3	107.1 (3)	H21A—C21—H21C	109.5
C24—C25—H25	126.4	H21B—C21—H21C	109.5
N3—C25—H25	126.4		

### Hydrogen-bond geometry (Å, °)

**Table:**

*D*—H···*A*	*D*—H	H···*A*	*D*···*A*	*D*—H···*A*
O1—H1···O6^i^	0.82	1.76	2.575 (3)	172
O1W—H1A···O6	0.86	1.90	2.747 (3)	168
O1W—H1B···O5^ii^	0.85	1.98	2.743 (3)	149
N4—H1C···O4^iii^	0.98	1.75	2.679 (4)	158
N2—H2···O1W^iv^	1.07	1.59	2.651 (4)	172
O8—H8···O3^v^	0.82	1.81	2.586 (3)	157

Symmetry codes: (i) *x*+1, *y*, *z*;  (ii) *x*, −*y*+1/2, *z*+1/2; (iii) *x*−1, −*y*+1/2, *z*−1/2; (iv) *x*−1, *y*, *z*; (v) −*x*+2, *y*+1/2, −*z*+3/2.
